# Acellular Dermal Matrices as an New Alternative for Treatment in Reproductive Organ Static Disorders: A Pilot Study

**DOI:** 10.3390/jcm13061550

**Published:** 2024-03-08

**Authors:** Marcin Sadłocha, Kaja Skowronek, Wojciech Łabuś, Jakub Staniczek, Maisa Mansar-Dyrbuś, Rafał Stojko

**Affiliations:** 1 The Chair and Clinical Department of Gynecology, Obstetrics and Oncological Gynecology, The Medical University of Silesia in Katowice, Markiefki 87, 40-211 Katowice, Poland; kskowronek@sum.edu.pl (K.S.); jstaniczek@sum.edu.pl (J.S.); maisamanasar@gmail.com (M.M.-D.); rstojko@sum.edu.pl (R.S.); 2 Dr. Stanislaw Sakiel Centre for Burn Treatment in Siemianowice Slaskie, Jana Pawla II str. 2, 41-100 Siemianowice Slaskie, Poland; wojciech.labus@clo.com.pl

**Keywords:** pelvic organ prolapse, polypropylene mesh, acellular dermal matrix, static disorders, allogenic implant

## Abstract

**Background:** The study aimed to evaluate the clinical effects of utilizing acellular dermal matrix (ADM) for treating pelvic organ prolapse. The motivation behind exploring a new treatment method stems from the limited efficacy of current surgical options, which are often associated with side effects. **Methods:** Ten patients with reproductive organ prolapse underwent surgery at the Chair and Department of Gynecology, Obstetrics, and Gynecological Oncology in Katowice. ADM was used as a support material, with eight patients receiving double TOT and two undergoing a six-point fixation mesh procedure. Pelvic organ prolapse was evaluated pre-operatively and one month post-surgery using the Pelvic Organ Prolapse Quantification (POP-Q) System. General medical history and complaints were assessed using the short form (PFDIQ-SF20). The study included ten patients aged 39 to 71 (mean: 63.6 years), all with a history of at least one vaginal delivery (mean of two). None had undergone a cesarean section. Four patients exhibited POP-Q 3, and five had POP-Q 2. **Results:** The mean PFDIQ-SF20 score before surgery was 70.6 points. No major complications occurred during or after surgery. One patient experienced a vaginal fungal infection and an allergic reaction to sutures. Post-operation, ailments reduced by an average of 60.76 points, with five patients reporting no complaints. **Conclusions:** ADM emerges as a material of interest for gynecological surgery, with initial reports highlighting its effectiveness and optimistic safety profile. Further research is warranted to explore its potential as a promising option in pelvic organ prolapse treatment.

## 1. Introduction

Pelvic organ prolapse (POP), frequently coexisting with stress urinary incontinence (SUI), is a prevalent condition among older women. The decline in pelvic floor muscles and connective tissues can result in the descent of pelvic organs. Each occurrence necessitates a personalized examination by a gynecologist for effective patient treatment and the prevention of potential serious side effects [1].

According to numerous studies, risk factors for pelvic organ prolapse (POP) encompass obesity, the number of deliveries, age, family history, the presence of constipation, and previous hysterectomy. Careful consideration of reproductive organ anatomy is crucial, as factors such as levator detachment, the preoperative stage of reproductive organ prolapse, and hiatus surface may elevate the risk of symptom recurrence following surgical treatment [2,3,4]. While vaginal delivery stands out as a significant and well-known risk factor for POP, the age at the last delivery and operative delivery do not appear to be associated with an increased risk. The impact of pregnancy itself and undergoing a cesarean section is also not definitively established. The risk of POP escalates with age, with a noteworthy finding that young age increases the risk of recurrence after surgical treatment. There is a significant association with urgency and mixed urinary incontinence, while surgical urinary incontinence, stress urinary incontinence, and other forms of urinary incontinence do not exhibit a significant association with primary POP. The impact of a previous hysterectomy remains uncertain. In primary POP, risk factors identified include levator defects and genital hiatus on transperineal ultrasound. Both unilateral and bilateral detachment, compared with no detachment, are significant risk factors. For POP recurrence, only levator defects show a significant association. It is essential to highlight modifiable risk factors resulting from lifestyle choices. An increased BMI or expanded waist circumference is a crucial risk factor for primary POP. However, moderate physical activity has a protective effect. Heavy weight lifting exercises have not been proven to increase the risk of POP [4].

Traditional methods of treating POP involve abdominal and vaginal correction. While these approaches generally result in good overall satisfaction rates, they are associated with a relatively high recurrence rate, reaching up to 30%. The quest for improved methods reflects the commitment to enhancing the quality of life for individuals dealing with pelvic organ prolapse. The new methods of treating these disorders are still being sought. Almost two decades ago, the use of a synthetic vaginal mesh became an alternative to the known techniques of using the patient’s native tissues in the treatment of reproductive organ static disorders. This method gained interest very quickly after its introduction to the market and enjoyed great support due to its positive effects, which resulted in the widespread use of these implants [1].

The prevalence of pelvic organ prolapse (POP) and urinary incontinence poses a significant challenge for healthcare systems, particularly in developed countries. The following are some key points highlighting the impact of these conditions on both individuals and the healthcare system: operations for static disorders in reproductive organs constitute a substantial portion, accounting for 20% of all gynecological operations in developed countries. These disorders may affect up to 50% of women who have given birth, emphasizing the widespread nature of pelvic organ prolapse and urinary incontinence. Approximately eleven percent of women below the age of 80 are anticipated to undergo surgery for different levels of pelvic organ prolapse or urinary incontinence. Notably, every third patient may necessitate more than one surgical procedure. Treatment choices encompass the utilization of autologous native tissue, the implantation of synthetic prostheses, and, frequently, the application of materials like polypropylene or biological substances. The choice of materials for surgical interventions reflects the diversity of available options, ranging from the patient’s own tissues to synthetic or biological materials. Particular groups of cancer patients, as a result of aggressive treatments for their underlying diseases, often face complications. While these treatments offer an opportunity for a longer life, they can be associated with issues such as pelvic organ prolapse and urinary incontinence [5,6].

However, soon after the use of vaginal mesh for reconstruction surgery in POP, the first serious complications were reported by a larger number of gynecologists. During the gynecological surgery congress of the Forum of Operative Gynecology in Berlin in 2007, over 400 known complications that may require difficult revision surgery were presented. The U.S. Food and Drug Administration (FDA) first issued a warning in 2008 and issued information about the danger in 2011, 2012 and 2013 [7,8].

The use of acellular dermal matrix (ADM) in tissue engineering presents a promising avenue, particularly in comparison to synthetic materials, due to its biocompatibility and the potential for fewer side effects. The following are some key points regarding the use and advantages of ADM. ADM is a type of cell-free tissue transplant, and its use is proposed to exclude unfavorable phenomena associated with synthetic materials. ADM is characterized by high biocompatibility, suggesting that it is well tolerated by the body. This property is crucial in minimizing adverse reactions and improving the overall safety profile. The method’s core involves utilizing the extracellular matrix of allogeneic dermis as a scaffold, offering mechanical protection against in vivo forces until the transplanted ADM seamlessly integrates into the body. This aspect is recognized as a significant advantage of the technique. Given that the skin is rich in collagen, it serves as a valuable source for biomaterials employed in tissue engineering. ADM, derived from allogeneic human dermis, preserves these advantageous properties. The process of creating ADM entails eliminating cells from allogeneic human dermis, resulting in a cell-free, collagen-rich, and non-immunogenic mesh. The acellular skin matrix (ADM) or acellular skin graft (ADG) obtained through this process can be de novo revitalized by autologous cells. This characteristic makes it a potential stimulant for natural mechanisms of regeneration and reconstruction. ADMs are typically created through a multi-stage application process, often involving the use of proteolytic enzymes on allogeneic human skin obtained from a deceased donor. The utilization of ADM in tissue engineering, with its cell-free and collagen-rich nature, represents a promising alternative to synthetic materials. Its biocompatibility, ability to provide mechanical protection, and potential for de novo revitalization by autologous cells contribute to its attractiveness in the field of regenerative medicine and surgical applications. The multi-stage creation process involving allogeneic human dermis reflects a meticulous approach to harnessing the benefits of this biomaterial for natural regeneration and reconstruction mechanisms [9].

Additionally, a limited inflammatory response of ADM has been substantiated by numerous scientific studies. Studies indicate that the immune response is primarily targeted against cell membrane proteins and lipids. Employing a method that involves removing cells from tissues appears promising as it aims to hinder the induction of an immune response in the patient’s body after implantation. Hence, the extraction of cellular components aims to minimize the immunologically induced inflammatory process, potentially alleviating the biodegradation of the transplanted bioprosthesis. In the study conducted by Holl et al.’s team [10], an analysis was performed on the impact of various methods of processing skin taken during abdominoplasty on the immunogenicity of hADM. The assessment also included the immunomodulatory properties of hADM and the influence of immune system cells on the structure of hADM. Three distinct decellularization methods were employed: the use of an ionic detergent (sodium dodecyl sulfate; SDS, in hADM 1), a non-ionic detergent (Triton X-100 in hADM 2), or a combination of recombinant trypsin and Triton X-100 (in hADM 3). Peripheral blood mononuclear cells (PBMCs) were isolated from the blood of healthy donors, and freshly isolated cells were incubated with the produced hADMs. Immunogenicity was assessed based on a T cell proliferation assay using flow cytometry, while the in vitro immunomodulatory potential was evaluated using multicolor flow cytometry and enzyme-linked immunosorbent assays. The impact of immune system cells on the collagen structure of the produced matrices was analyzed using confocal microscopy. The matrices under analysis displayed varying degrees of immunogenicity. hADM 1 induced low T cell proliferation without significant changes in the cytokine profile. In contrast, hADM 2 and 3 demonstrated higher immunogenicity compared to hADM 1. Subsequently, the activation level and phenotype of T lymphocytes and monocytes were assessed. Interestingly, none of the newly analyzed hADMs influenced the quantitative composition of selected T cell populations after 3 days of incubation. However, notable changes were observed in the percentage of monocyte subpopulations, specifically increased maturation towards cells with anti-inflammatory and pro-angiogenic potential. These changes were particularly significant after incubation of cells in the presence of hADM 1. Finally, the collagen structure of hADM after incubation with immune cells was examined, revealing the degradation of collagen IV and its colocalization with PBMC.

In conclusion, the study demonstrated that abdominoplasty skin is suitable for producing hADM. Additionally, the use of an ionic detergent in the processing of skin collected during abdominoplasty resulted in hADM with low immunogenicity and immunomodulatory properties. In another study by Groth et al. [11], three different decellularization protocols were used for skin treatment, namely 1M NaCl and sodium dodecyl sulfate (SDS, in ADM1); 2M NaCl sodium dodecyl sulfate (SDS, in ADM1); and a combination of recombinant trypsin and Triton X-100 (in hADM 3). The efficiency of decellularization and the structure of ADM were assessed using histochemical and immunochemical staining. The results indicated that all three proposed decellularization methods effectively removed cellular components from the skin after abdominoplasty. However, significant differences were found in the presence of human leukocyte class I antigen (HLA class I ABC), Talin 1/2, and chondroitin sulfate proteoglycan (NG2). Moreover, the study demonstrated that the protocols used differently influenced the behavior of collagen types I, III, IV, and VII. The dressing (ADM1) improved the kinetics of wound closure and scar maturation in the proliferation and remodeling phases of wound healing [11].

Our study aimed to assess the clinical effects of employing acellular dermal matrix (ADM) in pelvic organ prolapse treatment. The motivation to explore a new treatment approach stems from the incomplete efficacy of existing surgical options. Procedures involving synthetic materials often present various side effects, including infections, vaginal erosion, or dyspareunia. Conversely, operations using native tissue are frequently insufficiently durable.

## 2. Materials and Methods

The study was conducted in accordance with the principles and guidelines set forth in the Helsinki Congress (revised in 2013) and the Istanbul Declaration, emphasizing the importance of ethical standards in medical research. The consent of the bioethics committee no. KNW/0322/KB1/100/22 was obtained.

The patients were informed about the type of material used, which was a tissue graft from a deceased donor. The possibilities, limitations, and risks associated with the use of ADM were thoroughly discussed with them. They provided signed consent for both the surgery and the transplant.

### 2.1. Tissues Preparation

Donors of allogeneic skin were included in the study in accordance with the Polish transplant law implementing Directive 2004/23/EU, Commission Directive 2006/17/EC, and Commission Directive 2006/86/EC. All activities related to the work with the tissue material were performed in Tissue Establishment TE of The Centre for Burn Treatment in Siemianowice Śląskie, Poland CLO in a clean room class GMP A (ISO 4.8) provided by a laminar air flow cabinet in the area of class GMP C (ISO 7).

### 2.2. Human Allogeneic Skin Procurement

The allogeneic skin is obtained from a deceased donor by a trained member of the procurement team. The procurement is conducted under aseptic conditions to minimize the risk of contamination. A battery dermatome (Acculan III, Aesculap) is used to harvest skin at the desired thickness (0.2–0.5 mm). The harvested allogeneic skin is transported to the CLO Tissue Bank following established standard operating procedures. The decellularization process involves a 1 h incubation of human dermis in a 2.4 U/mL Dispase II enzyme solution, followed by a 24 h incubation in a 0.05% trypsin solution. These enzymatic treatments are likely designed to remove cellular components, leaving behind the extracellular matrix (ECM). The prepared ADMs are sterilized using electron beam radiation. Electron beam radiation at 35 kGy is a common method for sterilizing biological materials, as it effectively kills or inactivates microorganisms without leaving residues. The final product is an acellular dermal matrix which is devoid of cellular components but retains the structural and biochemical properties of the native dermis.

### 2.3. Patients Qualified for ADM Treatment Method

**Figure 1:**
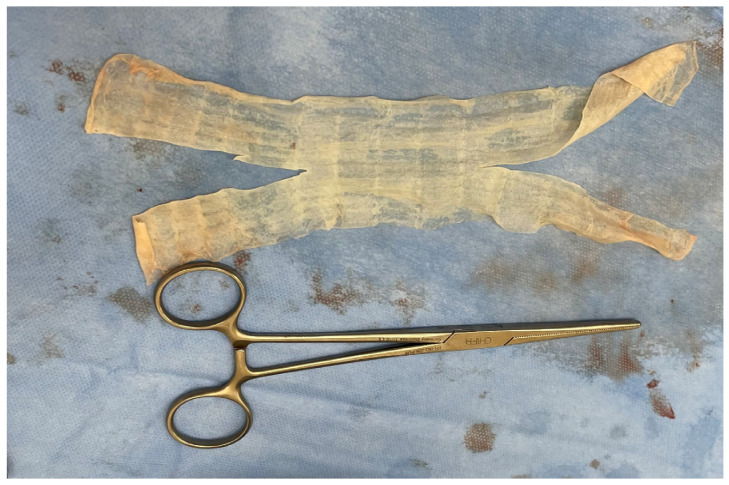
Acellular dermal matrix—mesh template.

**Figure 2:**
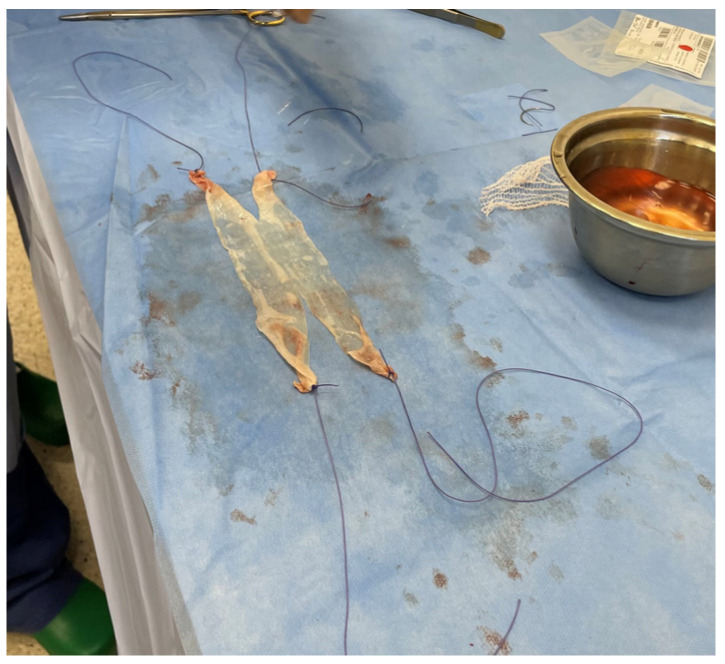
Acellular dermal matrix—final implant.

**Figure 3:**
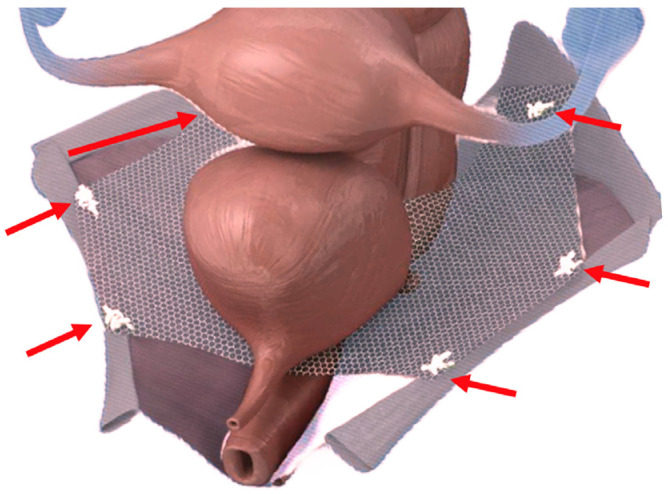
Acellular dermal matrix—six-point mesh concept.

**Figure 4:**
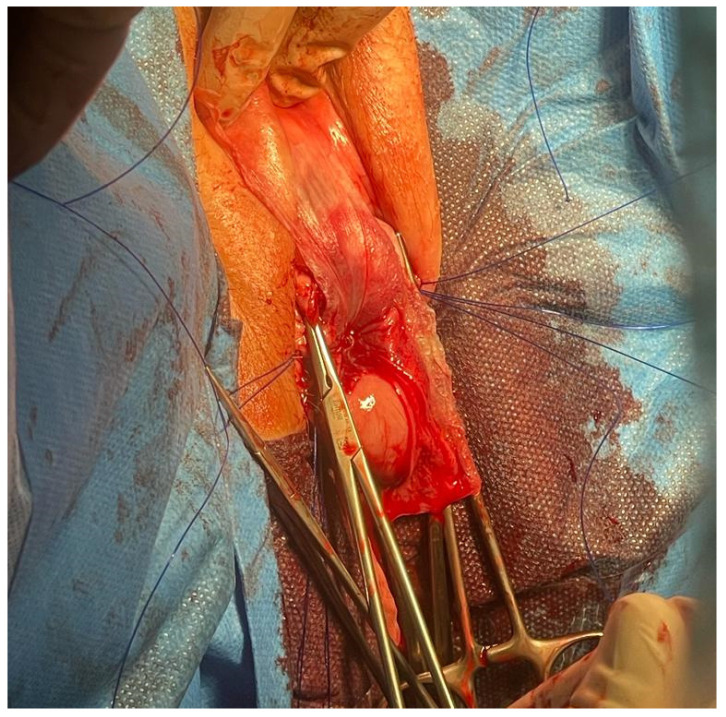
Acellular dermal matrix—intra-corporal location.

Ten patients with reproductive organ prolapse underwent surgery at the Chair and Department of Gynecology, Obstetrics, and Gynecological Oncology in Katowice. The study spanned from November 2022 to September 2023. All surgeries were tailored to the specific type of defect, utilizing an acellular dermal matrix (ADM) as a support material. In eight patients, the matrices were implanted as a double TOT, while in two patients, a six-point fixation mesh was employed (see Figure 1, Figure 2, Figure 3 and Figure 4). The application of the six-point mesh was chosen for cases involving a defect in the lateral anterior compartment, where the prolapse affected both the anterior vaginal wall and the cervix.

Before the surgery and six months post operation, we evaluated genital prolapse using the Pelvic Organ Prolapse Quantification (POP-Q) System. Additionally, we conducted a general medical history review and assessed complaints using the short form of the pelvic floor distress inventory questionnaire (PFDIQ-SF20).

## 3. Results

The provided information outlines the demographic details, surgical treatment, and outcomes of ten patients who underwent surgery for pelvic organ prolapse (POP).

Ten patients participated in the surgical treatment. Patient ages ranged from 39 to 71 years. The mean age of the patients was 63 years. Every patient had at least one vaginal delivery. The average number of vaginal deliveries per patient was two. None of the patients had a cesarean section. Pelvic Organ Prolapse (POP) Assessment: Four patients had prolapse assessed as POP-Q stage 3. Six patients had prolapse assessed as POP-Q stage 2.

Pelvic Floor Distress Inventory Short Form 20 (PFDIQ-SF20) Score: the mean PFDIQ-SF20 score before surgery was 67. Six months after surgery, the pelvic floor distress inventory short form 20 (PFDIQ-SF20) score decreased, indicating an improvement in symptoms. On average, postoperative scores reduced by 60 points. (Table 1). There were no major complications reported during or after surgery. One patient developed a vaginal fungal infection. Another patient experienced an allergic reaction to intradermal sutures. After the surgical intervention using ADM, the patients experienced a reduction in symptoms. Five patients did not report any complaints postoperatively.

There was no statistical relationship between the number of deliveries and BMI and the PFDIQ-20 questionnaire result before the procedure (*p* > 0.05).

The surgical treatment for pelvic organ prolapse in these ten patients, with a focus on vaginal deliveries and the absence of cesarean sections, resulted in an improvement in symptoms and a reduction in the PFDIQ-SF20 score. The absence of major complications is notable, although minor complications such as a vaginal fungal infection and an allergic reaction to sutures were reported. The overall outcome suggests a positive impact on the patients’ well-being and a reduction in pelvic floor distress.

## 4. Discussion

The use of ADM in medicine is an increasingly popular issue. Last year, 278 publications appeared on this subject in the PubMed database alone. Such a large amount of work provides us with information regarding the safety and effectiveness of the use of ADM. The most common areas of ADM use include breast and muscle reconstructive surgery, periodontal treatment, and the treatment of wounds and burns. The interest in the use of ADM results from the insufficient effectiveness of available synthetic materials used in this type of diseases and their complications.

Seifalian et al. demonstrated in their analysis that most clinical studies evaluating polypropylene pelvic (PP) meshes deemed them biocompatible and non-toxic, despite the associated risk of complications, particularly mesh exposure. They highlighted the limited number of studies assessing the mechanical properties of PP mesh implants and the potential mismatch of viscoelastic properties with the surrounding native tissue. The majority of clinical trials, often of a short duration (less than three years), tend to overlook long-term complications. The ideal mesh should possess biocompatibility, non-toxicity, antibacterial properties, mechanical characteristics akin to the surrounding pelvic tissue, and the ability to withstand high pressure without cracking or failure [12].

A recent publication comparing commercially available synthetic meshes and their associated events found that the most frequently reported complications were pain, erosion and infection. Among transvaginal implants, <10% of Uphold Lite (Boston Scientific, Marlborough, MA, USA) reports contained pain or erosion vs. >90% of Prolift/Prolift+M (Ethicon, Raritan, NJ, USA; *p* < 0.001). Researchers emphasized the different properties and side effect profiles of individual meshes and signaled the need to maintain comprehensive registers of such reports [13].

Comparison of Human ADM and non-absorbable polypropylene mesh in rabbits showed that after 180 days, the incidence of vaginal mesh extrusion was higher in the PP group (33% vs. 0%, *p* = 0.015). Full integration of vaginal grafts occurred more often in the hADM group (35% vs. 0% in the PP group, *p* = 0.014). In the PP group, the infiltrates were focal, while in the hADM group, the infiltrates were diffuse. Biomechanical analysis showed that hADM was characterized by lower resistance to stress, stiffness and elasticity. To sum up, hADM is associated with fewer clinical complications and better integration with surrounding tissues, which unfortunately may translate into a reduction in its biomechanical properties 6 months after implantation [14].

Due to complications occurring after the use of synthetic meshes, some countries have banned their use. This has consequences. An analysis of POP surgery in Australia published in 2023 observed a decline in the overall number of POP procedures performed over the past 15 years. However, recently, there has been an increase in the number of interventions in the private sector, and the use of laparoscopic or abdominal POP repair has increased [15]. It is worth emphasizing that this method of surgery is not applicable to all types of POP defects and is more burdensome than transvaginal operations.

In our opinion, urogynecology remains an area where the potential of ADM has not been sufficiently exploited. A significant limitation in our search for appropriate literature was the small number of publications, small groups of patients, and the frequent use of porcine ADM, which, although it has similar properties, is not the same product. There are isolated reports of the use of ADM in the treatment of uterine prolapse in women.

At the Peking University People’s Hospital, 20 patients with vaginal wall prolapse were operated on. In all of them, the lowering affected the anterior vaginal wall, and in 17 of them, there was also a prolapse in the posterior vaginal wall. No erosion or infection was observed in any of the operated patients during the follow-up period lasting from half a year to a year. The symptoms recurred in four patients and occurred after 6 months. In three patients, the re-lowering of the reproductive organs was grade I, and in one, grade II. Patients did not report any other complaints [16].

Similar results were shown by Ward et al. In his retrospective study on 33 patients with primary or recurrent vaginal prolapse in grades II to IV. Full correction of the anatomical defect was achieved in 64% of patients. In the remaining group (13 patients), 92%, despite incomplete correction, had no symptoms. None of the 21 sexually active women reported postoperative dyspareunia. Intra- and postoperative complications included one infection, one cystotomy, and one anterior vaginal hematoma, most likely caused by heparin therapy. No erosion or rejection of material was observed [17].

The use of ADM in urogynecology is not limited only to surgeries reducing the prolapse of the reproductive organ. They have also been successfully used in reconstructive surgeries. In 2016, Bhavsar et al. used the ADM in a 51-year-old patient after abdominoperineal resection and end colostomy due to rectal adenocarcinoma. During the operation, the posterior vaginal wall was partially removed. The condition after previous radiotherapy due to cervical cancer and the resulting narrowing made it impossible to restore the continuity of the vagina using native tissues. The time for complete vaginal healing was approximately 7 months. The posterior vaginal wall did not differ morphologically from the normal vagina. Three years after the surgery, narrowing of the vaginal vaults and atrophy of the mucous membrane occurred [18].

You et al. compared the standard method of treating vaginal–rectal fistula with surgery using ADM. The study group included 12 people and the control group 10. Full therapeutic success was achieved in 11 patients, and reoperation was necessary in one case. The use of ADM showed greater effectiveness and less traumatization [19]. Some 3 years earlier, Zhu et al. used ADM to create a vagina in 53 patients with Mayer–Rokitansky–Küstner–Hauser (MRKH) syndrome. The control group consisted of healthy women of the same age as the study group. Anatomical success was considered in all patients, i.e., vaginal length ≥ 8 cm and width of at least two fingers. In 32 patients who were sexually active, 75% obtained results in the FSFI (Female Sexual Function Index) questionnaire similar to those of the control group (26.7 ± 3.5 vs. 25.6 ± 7.4, *p* = 0.46). Postoperative vaginal granulomatous polyps requiring outpatient removal developed in 11.3% of patients [20].

A similar study with a longer follow-up period (median 57 months) was published by Mao et al. In 2023, the most common postoperative complication was neovaginal granulomatous polyps (7 of 42, 16.7%). The vaginal length in the MRKH group was comparable to the vaginal length in the control group (8.04 ± 0.51 cm vs. 8.15 ± 0.46 cm, respectively). FSFI scores were similar in the MRKH group (26.54 ± 3.44) and the control group (26.80 ± 2.23). Patients with MRKH syndrome can therefore achieve long-term satisfactory results both anatomically and functionally after vaginoplasty with ADM, comparable to the results of healthy control women [21].

Wang et al. In 2019 used ADM for vaginoplasty in 16 patients with cervical cancer treated with surgery and radiotherapy. In the first stage, surgery was performed, and then the patients used vaginal dilators for 6 months. Assessment was made after 12 months. The vaginal width increased significantly from 1.31 ± 0.4 cm before the procedure to 4.13 ± 0.43 cm after the procedure (*p* = 0.034). The vaginal length also increased from 5.97 ± 0.59 cm to 9.25 ± 0.66 cm (*p* < 0.001). Most patients (75%) declared a satisfactory sex life [22]. ADMs are also used as a tissue patch in the treatment of vesicovaginal fistulas. In 2007, Yang et al. used a retroperitoneal matrix to repair four cases of vesicovaginal fistulas. During the follow-up period lasting up to 12 months, none of the patients reported a recurrence of urine leakage [23]. Good results were also achieved by a team of researchers from China, who performed this procedure via vaginal natural orifice transluminal endoscopic surgery in 2021 [24].

In recent years, there has been a resurgence in the use of native tissues for surgery, both in traditional and modified forms. This approach aims to mitigate the typical side effects associated with synthetic meshes. An illustrative example is the practice of colpocleisis, a long-standing and safe method that, regrettably, renders vaginal intercourse impossible. Despite this limitation, it proves to be an effective approach, significantly alleviating reported patient symptoms [25]. Unfortunately, relapses are observed even following this type of treatment. Risk factors for recurrence include a wide pre- and postoperative genital hiatus and an extended postoperative total vaginal length (TVL). The likelihood of recurrence can be minimized through the careful selection of patients [26].

Among the limitations of this research are the limited number of patients resulting from the pioneering nature of the study and the possibility of financing ADM. Due to the optimistic results obtained in the initial group of patients, we plan to expand the study to a total of 150 patients operated on within the next 5 years. The control group will consist of patients operated on with the currently available method using synthetic mesh. A more detailed statistical analysis is necessary for a better interpretation of results. We will enhance the statistical analysis section to provide a clearer understanding of the observed statistical significance and effect sizes when the study will be extended. Due to the paucity of studies comparing ADM and currently available surgical methods using native tissues or synthetic materials, we plan to expand our study group and compare it with a control group, which will consist of patients after the insertion of a synthetic six-point mesh for the treatment of genital prolapse.

## 5. Conclusions

One of the key advantages of ADM is its ability to induce a limited inflammatory response. This can be beneficial in surgical applications, as excessive inflammation can lead to complications. The limited inflammatory response may contribute to a reduction in complications, particularly when compared to synthetic mesh materials. Complications like vaginal erosions are less likely to occur with ADM. ADM is biocompatible, meaning it is well tolerated by the body. This is crucial for its use in surgical procedures, as it reduces the risk of adverse reactions or rejection. Compared to autologous transplants (using the patient’s own tissue), ADM offers the advantage of shortening the surgical time and reducing the number of procedures required. This can lead to quicker and potentially less complex surgeries. It is important to note that while initial reports may be optimistic, ongoing research and long-term studies are necessary to further validate the safety and efficacy of ADM in various surgical applications. Additionally, individual patient factors and the specific surgical context may influence the outcomes. Surgeons and healthcare professionals should typically consider a range of factors when choosing the most appropriate surgical mesh for a given case.

## Figures and Tables

**Table 1:** Study group [*n* = 10].

*n*	AGE	Vaginal Birth	(PFDIQ-SF20) Before	POPQ Before	PFDIQ after 6 msc	POPQ after 6 msc	Bedsore	Erosion
1	63	2	65	3	6.25	1	0	0
2	69	2	65	3	0	2	0	0
3	70	5	25	3	0	0	0	0
4	67	2	47	2	0	0	0	0
5	67	2	103	2	25	1	0	0
6	39	3	25	2	0	0	0	0
7	68	1	91	3	12	1	0	0
8	60	2	70	2	0	0	0	0
9	71	2	112	2	33	0	0	0
10	63	3	109	2	25	0	0	0

## Data Availability

Data available on request due to restrictions (General Data Protection Regulation, also known as the Personal Data Protection Regulation, GDPR or GDPR—an EU regulation containing provisions on the protection of natural persons with regard to the processing of personal data and provisions on the free flow of personal data. Effective date: 25 May 2018). The data presented in this study are available on request from the corresponding author (msadlocha@sum.edu.pl).

## Abbreviations

ADM	acellular dermal matrix
TOT	trans-obturator tape
POP-Q	Pelvic Organ Prolapse Quantification
PFDIQ-SF20	Pelvic Floor Distress Inventory (short form)
POP	pelvic organ prolapse
FDA	Food and Drug Administration
ADG	acellular skin graft
ECM	extracellular matrix
PP	polyprepylene pelvic (meshes)
MRKHS	Mayer–Rokitansky–Kuster–Hauser syndrome
TVL	total vaginal length
